# The Genetically Homogeneous Population Structure of *Evynnis cardinalis* Along the Chinese Coast Revealed by Whole Genome Sequencing

**DOI:** 10.1111/eva.70272

**Published:** 2026-05-28

**Authors:** Sailan Liu, Yan Gao, Xingboyu Zhang, Xinrui Long, Qilin Gutang, Hancheng Zhao, Jianqing Lin, Wenhua Liu

**Affiliations:** ^1^ Guangdong Provincial Key Laboratory of Marine Biotechnology, Institute of Marine Sciences Shantou University Shantou China; ^2^ International Joint Research Center for Marine Ecological Protection and Disaster Prevention Shantou University Shantou China; ^3^ State Key Laboratory of Genetics and Development of Complex Phenotypes, School of Life Sciences Fudan University Shanghai China

**Keywords:** adaptive evolutionary, *Evynnis cardinalis*, genome, population genetic structure

## Abstract

*Evynnis cardinalis* is a warm‐water demersal fish inhabiting the northwestern Pacific coastal waters. Previous studies based on limited molecular markers and morphological traits have yielded inconsistent conclusions regarding its phylogenetic position and population differentiation. To address these uncertainties, we assembled a high‐quality chromosome‐level genome using PacBio HiFi and Hi‐C technologies, and then clarified its close phylogenetic relationship with *Pagrus major* . At the population genomic level, genome‐wide single nucleotide polymorphism (SNP) analysis detected 8,643,907 high‐quality SNPs from three geographically distinct populations (Shantou, Zhuhai, and Hainan). The results revealed weak population structure and extremely low genetic differentiation among the three geographic populations, a pattern consistent with high genetic connectivity in the ocean. However, subsequent analysis showed that chromosome 8 had the most candidate genes for genomic variation among populations, involved in cell structure, energy metabolism, and DNA repair. It suggests that certain genomic regions may provide potential targets for future studies on environmental responses in complex nearshore benthic environments despite the overall population genetic homogeneity. Our study not only provides the first chromosome‐level genome assembly but also clarifies the taxonomic status and single population genetic structure of *E. cardinalis* in Chinese coastal waters at genome‐wide level. These findings are expected to facilitate the biodiversity conservation and sustainable management of this commercially important species.

## 1 Introduction

Clarifying phylogenetic relationships and population differentiation mechanisms is one of the core themes of evolutionary biology. By integrating high‐fidelity long‐read sequencing (e.g., PacBio HiFi) with Hi‐C chromatin conformation capture technology to assemble chromosome‐level reference genomes, highly continuous genome assembly can be achieved. This facilitates the identification of genome‐wide orthologous sites among species, significantly enhancing the robustness and resolution of phylogenetic inference (Gao et al. 2024; He et al. 2024). For morphologically similar and widely distributed fish species, traditional genetic markers including Cytochrome oxidase I (*COI*) and Cytochrome b (*Cytb*) may provide limited information for resolving recent divergence, complex lineages or Incomplete Lineage Sorting (ILS) (Degnan and Rosenberg 2009). In contrast, genome‐wide data provide higher resolution and accuracy for phylogenetic relationships. In particular, whole‐genome resequencing (WGS) can generate large numbers of single nucleotide polymorphisms (SNPs), enabling fine‐scale inference of genetic structure and demographic history while reducing the risk of misclassification (Pappas et al. 2023), and has shown important application value in population delineation in aquatic species (Tang et al. 2024; Liu et al. 2025).

For fisheries management, accurate population structure information is essential for scientifically defining fisheries management units, which directly affects the accuracy of stock assessment and the effectiveness of management strategies. Misclassifying genetically homogeneous populations into multiple management units may lead to deviation in resource assessment, duplication of conservation measures, and increase in management costs (Reiss et al. 2009). Conversely, treating them as a single management unit can optimize breeding and releasing, and quota allocation, thereby reducing costs and promoting sustainability (Funk et al. 2012). WGS enables the detection of local adaptive genes, revealing population‐specific responses to environmental pressures (Xu et al. 2020; Liu et al. 2025). Overlooking such local adaptations may cause a single management model to underestimate part of ecological risks (Benestan et al. 2016). Therefore, combining high‐quality reference genome with genome‐wide variation data is of great practical significance for obtaining accurate phylogenetic relationships, population structure characteristics, and adaptive divergence, thereby offering actionable scientific support for formulating efficient and economical regional conservation strategies.

*Evynnis cardinalis* , a small‐sized, warm‐water, demersal species in the family Sparidae (order Perciformes), inhabits coastal waters at depths of 30–60 m in the northwestern Pacific (Chen and Qiu 2005). Its taxonomic status has been a controversial subject. Initially described as *Parargyrops edita* , it was later reclassified as *E. cardinalis* based on cranial and dental morphology (Cheng and Zheng 1987; Wu et al. 1999). Despite this taxonomic revision, controversy remains regarding the phylogenetic relationship between *Evynnis* and *Pagrus*. While some morphological studies support their differentiation, others highlight their skeletal and coloration similarities (Chen et al. 2014). And the similarities between the two genera were also supported by several genetic markers, including random amplified polymorphic DNA (RAPD) (Yang and Jiang 2006), Cytb (Xu et al. 2010), Displacement Loop (D‐loop) (Su et al. 2012), protein‐coding genes (PCGs) (Xia et al. 2008), and *COI* (Huang et al. 2017). These studies indicate that *Evynnis* and *Pagrus* exhibit interspecific differentiation, but the divergence evidence is not enough to support the separation at the genus level. Geographically, *E. cardinalis* is widely distributed along the southern East China Sea and the South China Sea, especially in important fishing grounds such as the Minnan–Taiwan shoal and the Beibu Gulf. However, the population structure of this species remains controversial. Morphometric analyses suggested the distinct populations between the Beibu Gulf and Taiwan Strait, possibly due to geographic barriers and habitat difference like the Leizhou Peninsula (Zhang and Cai 1983). In contrast to the morphological evidence, genetic studies have provided different insights. Multiple genetic studies using mitochondrial D‐loop (Gong 2007), Simple Sequence Repeat (SSR) (Bing Yang 2015), and *COI* (Xu et al. 2021) markers consistently reveal low genetic differentiation among populations, supporting a single, homogeneous population along the Chinese coast. This disagreement between morphological and genetic data highlights the need for genome‐wide evidence to re‐evaluate phylogenetic position and population structure.

In this study, we aim to assemble a chromosome‐level reference genome of *E. cardinalis* based on PacBio HiFi and Hi‐C technologies, and combine it with WGS data to address two questions: (i) clarifying its phylogenetic placement within Sparidae by analyzing its relationship between *P. major* , thereby providing genomic evidence for the long‐standing genus classification controversy; and (ii) revealing population genetic structure across Chinese coastal waters to explain conflicting conclusions based on morphological and traditional marker studies. This study will not only offer valuable genomic resources for future biological and genetic investigations of this species, but also provide new insights into its population structure, genetic diversity, and adaptive potential. Furthermore, this study will support future conservation and management of fishery resources.

## 2 Materials and Methods

### 2.1 Sample Collection and Preparation

**FIGURE 1:**
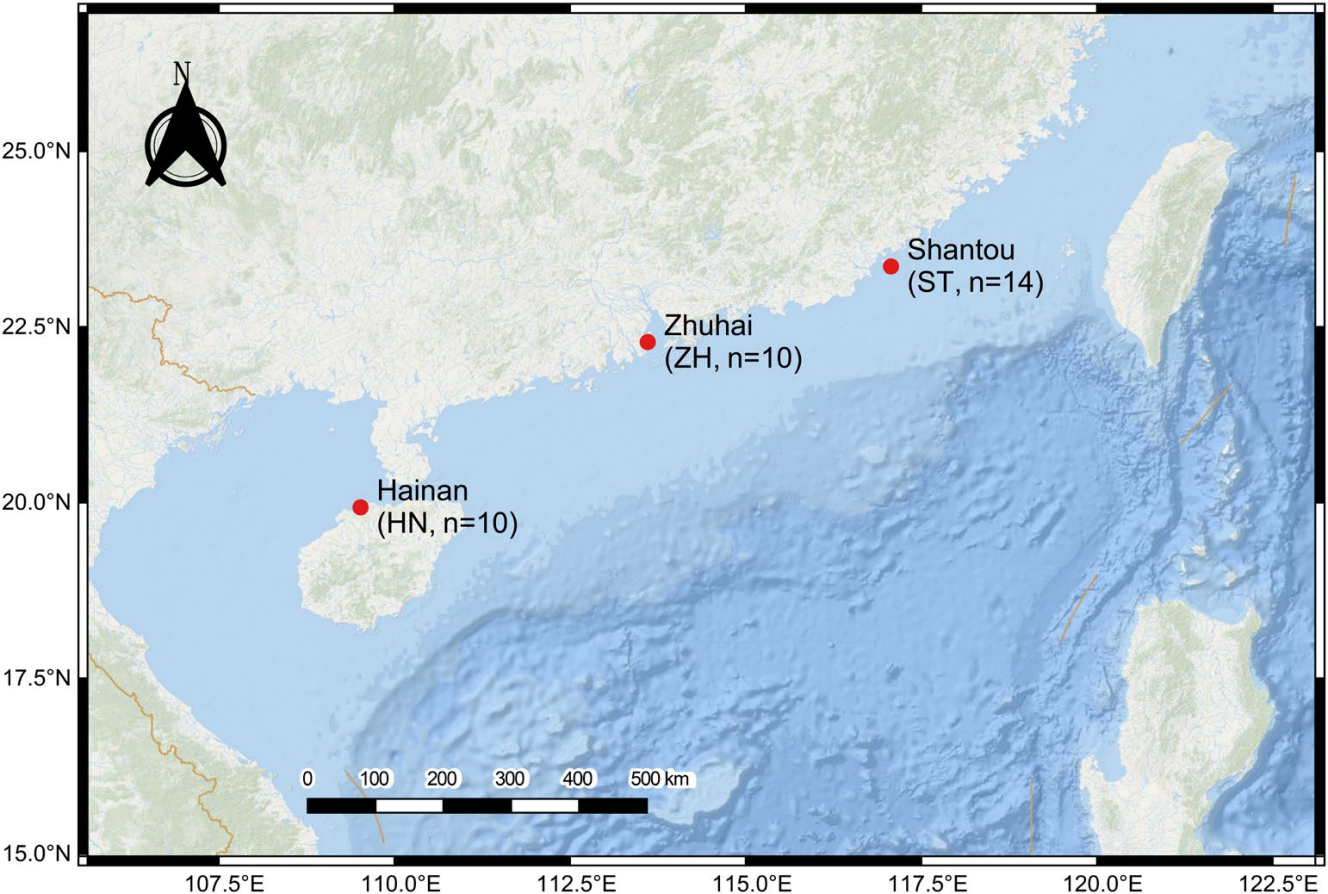
Map indicating the sampling regions (Shantou [ST], Zhuhai [ZH], and Hainan [HN]).

A wild individual of *E. cardinalis* was collected from the coastal waters near Nanao Island, Shantou, Guangdong Province, for chromosome‐level genome assembly. In addition, a total of 34 individuals were sampled from three coastal locations in Shantou (ST, *n* = 14), Zhuhai (ZH, *n* = 10), and Hainan (HN, *n* = 10) for WGS (Figure 1). All samples were transported to the laboratory under low‐temperature conditions. Muscle tissues were rapidly frozen in liquid nitrogen and stored at −40°C.

### 2.2 DNA Extraction and Library Construction

Approximately 700 ng of high‐quality genomic DNA (gDNA) extracted from muscle tissue was used to construct Illumina libraries (PE 150), sequenced on the Hiseq 6000 platform (Illumina, San Diego, CA, USA). Hi‐C libraries were constructed according the modified standard procedure: MboI digestion, enrichment with streptavidin beads, adapter ligation, and 12–14 PCR cycles, followed by Illumina PE 150 sequencing. HiFi libraries were prepared with the SMRTbell Express Template Prep Kit 2.0 (Pacific Biosciences, Menlo Park, CA, USA), targeting approximately 20 kb fragments generated using the g‐TUBE device (Covaris, Woburn, MA, USA), and sequenced on the PacBio Sequel II platform after repair, adapter ligation, and BluePippin selection. Stranded mRNA libraries were constructed from muscle RNA and sequenced on the Illumina Hiseq platform with 150 bp paired‐end reads.

### 2.3 Quality Control and Genome Survey

For Illumina and Hi‐C data, raw reads containing adapter, > 10% ambiguous bases (N), or > 20% low‐quality bases (Phred < 5) were removed. Clean short reads were used to estimate the genome size, heterozygosity, and repeat content with jellyfish 2.2.7 (Marçais and Kingsford 2011). And high‐quality Hi‐C data were used to assist in constructing chromosome‐level genome. PacBio Polymerase reads were processed using the ccs tool (min‐passes = 3, min‐rq = 0.99) to generated HiFi reads with quality scores above Q20. Transcriptome datasets included six muscle RNA‐seq datasets of *E. cardinalis* from NCBI (SRX7249650 to SRX7249655) combined with newly generated, all quality‐filtered with fastp v0.23.2 with default parameters (Chen 2023).

### 2.4 Genome Assembly and Quality Assessment

HiFi reads were assembled using HiFiasm v0.19.5‐r587 (Cheng et al. 2021) under default parameters. Hi‐C reads were aligned with juicer v1.6 (Durand et al. 2016), and scaffolding was constructed with 3D‐DNA v180922 (Dudchenko et al. 2017). Manual curation was performed using Juicebox v2.20.00, followed by reprocessing with 3D‐DNA to produce the final chromosome‐level genome assembly. Assembled quality was assessed with QUAST v5.2.0 (Gurevich et al. 2013) and BUSCO v5.1.2 with actinopterygii_odb10 database (Simão et al. 2015). Telomeric sequences at the ends of the 24 scaffolds were detected with quarTeT v1.1.6 to confirm the chromosomal‐level assembly (Lin, Ye, et al. 2023).

### 2.5 Repetitive Elements Identification and Genome Annotation

The species‐specific repeat library was constructed using RepeatModeler v2.0.1 (Flynn et al. 2020). The repeat elements were then identified using RepeatMasker 4.1.2‐p1 and Repbase database (Jurka et al. 2005). tRNAs were predicted using tRNAscan‐SE v2.0.9 (Chan et al. 2021), and rRNAs, snRNAs, and miRNAs were identified with Infernal v1.1.4 (Nawrocki and Eddy 2013) and the Rfam database (Griffiths‐Jones et al. 2003). Protein‐coding genes were annotated by *de novo* prediction, homology‐based prediction, and transcriptome‐based prediction methods. For gene prediction, previously available *E. cardinalis* transcription sequences downloaded from NCBI (SRX7249650 to SRX7249655) were used as species‐specific homology evidence and aligned to the assembled genome to support gene prediction. The filtered RNA‐seq reads were aligned with TopHat v2.1.1 (Kim et al. 2013) after genome indexing with Bowtie2 v2.4.2 (Langmead and Salzberg 2012). Intron–exon structures were identified using Cufflinks v2.2.1 (Trapnell et al. 2010) as EST evidence. MAKER v3.01.03 was iteratively run for six rounds, including four‐round optimized SNAP models, Augustus (trained on zebrafish), and GeneMark‐ET (Holt and Yandell 2011). To evaluate the Annotation completeness was assessed with BUSCO v5.1.2 (Simão et al. 2015) with the Actinopterygii_odb10 database.

### 2.6 Phylogenetic Analysis and Divergence Time Estimation

Genome assemblies and annotation files (GFF) of 10 representative bony species ( *Danio rerio* , *Takifugu rubripes* , *Oryzias latipes* , *Collichthys lucidus* , *Notolabrus celidotus* , *Oreochromis niloticus* , *Hippocampus comes* , *A. latus* , *P. major* , and *Sparus aurata* ) were downloaded from NCBI and Ensemble databases. Orthologous gene clustering was performed using OrthoFinder v2.5.4 (Emms and Kelly 2019) and clustered with the Markov Cluster Algorithm (MCL). Single‐copy orthologs were extracted for subsequent phylogenetic analysis. Protein sequences were aligned with MAFFT v7.475 (Katoh and Standley 2013), converted to codon alignments with ParaAT v2.0 (Zhang et al. 2012), and trimmed with trimAl v1.4.rev15 (Capella‐Gutiérrez et al. 2009). Phylogenetic tree was constructed with RAxML‐NG v1.0.2 based on the GTR + I + G4 model with 1000 bootstrap replicates (Kozlov et al. 2019). ASTRAL v5.7.3 was used to infer a consensus coalescent tree, with quartet support values assessing ILS or introgression (Zhang et al. 2018).

### 2.7 Genome Family Evolution

Gene family expansion and contraction (*p* < 0.05) in *E. cardinalis* were identified with CAFÉ v5.0 (Mendes et al. 2020). And Gene Ontology (GO) and Kyoto Encyclopedia of Genes and Genomes (KEGG) enrichment were analyzed using clusterProfiler (Wu et al. 2021). Species‐specific gene families were extracted from OrthoFinder results for functional enrichment analysis. Rapidly evolving genes were detected by comparing one‐ratio (model = 0, NSsites = 0) and two‐ratio (model = 2, NSsites = 0) models in PAML v4.9 with the CODEML module (Ziheng Yang 2007). For positively selected genes (PSGs), we applied the branch‐site model (model = 2, NSsites = 2), comparing the null model (fix_omega = 1, omega = 1) and the alternative model (fix_omega = 0, omega estimated), and considered genes with *p* < 0.05 and Bayes Empirical Bayes (BEB) posterior probability ≠ NA as PSGs. Both REGs and PSGs were functionally annotated through GO and KEGG enrichment analysis using clusterProfiler.

### 2.8 WGS and SNP Calling

WGS of 34 individuals was performed on the BGISEQ‐500 platform (PE 150). Raw reads were processed with the PALEOMIX v1.3.8 pipeline for adapter trimming and filtering (Schubert et al. 2014). Clean reads were then aligned to the newly assembled *E. cardinalis* reference genome with BWA v0.7.17‐r1188 and sorted with Picard‐tools v1.118 (Li et al. 2009). Mapping statistics, including genome coverage and depth, were assessed using SAMtools v0.1.20 (Li et al. 2009). Variants were called with GATK v4.3.0.0 HaplotypeCaller, merged into a joint VCF file, and filtered (QD < 2.0, MQ < 40.0, FS > 60.0, SOR > 3.0, MQRankSum < −12.5, and ReadPosRankSum < −8.0) (McKenna et al. 2010). Further quality control was applied to retain only high‐quality biallelic SNPs with QUAL > 40, minor allele frequency (MAF) ≥ 0.05, and a call rate ≥ 95% (‐‐maf 0.05, ‐‐max‐missing 0.95). Finally, functional annotation was conducted using ANNOVAR (Wang et al. 2010) based on the annotation of the *E. cardinalis* reference genome.

### 2.9 Population Genetic Structure Analysis

Population genomic analyses were based on high‐quality SNPs. Genetic distances between individuals were calculated using VCF2Dis v1.47, and a Neighbor‐Joining tree (NJ) was built using PHYLIP v3.697 with 1000 bootstrap replications and visualized with ChiPlot (Xie et al. 2023). PCA was performed with Plink v1.90b6.21 and visualized in R (Chang et al. 2015). ADMIXTURE v1.3.0 (Falush et al. 2003) was run with *K* = 1–4, and cross‐validation error (CV error) was used to determine the optimal *K*.

To comprehensively analyze the genetic structure of *E. cardinalis* , we applied three complementary machine learning methods: Discriminant Analysis of Principal Components (DAPC), Random forest‐based Multidimensional Scaling RF‐MDS and t‐distributed Stochastic Neighbor Embedding (t‐SNE). In DAPC analysis, PCA reduced the SNP genotype matrix while retaining 95% of genetic variations (n_components = 0.95), *K*‐means with the elbow method determined the optimal number of clusters (*K* value), and linear discriminant analysis (LDA) maximized population differentiation. RF‐MDS used a 500 trees (n_estimators = 500) random forest model to generate the kinship matrix from leaf co‐occurrence frequencies, which was quantified with Hamming distance and visualized with MDS. t‐SNE was used to examine the genetic continuity, with perplexity set to 30 and Barnes‐Hut optimization for computational efficiency.

### 2.10 Population Genetic Diversity and Detection of Candidate Regions

Based on the high‐quality SNPs, nucleotide diversity (*Pi*), genetic differentiation (*F*_*ST*_), and Tajima's *D* were calculated using VCFtools v0.1.16 with a sliding window size of 100,000 bp and a step size of 10,000 bp (Danecek et al. 2011). And *F*_*ST*_ values in the top 5% of the genome‐wide distribution together with reduced *Pi* values in at least one population were identified as candidate windows. Adjacent or continuous windows were then merged into candidate regions. Tajima's *D* was used as supportive evidence for interpreting local selection signals.

## 3 Results

### 3.1 Genome Assembly and Annotations

**FIGURE 2:**
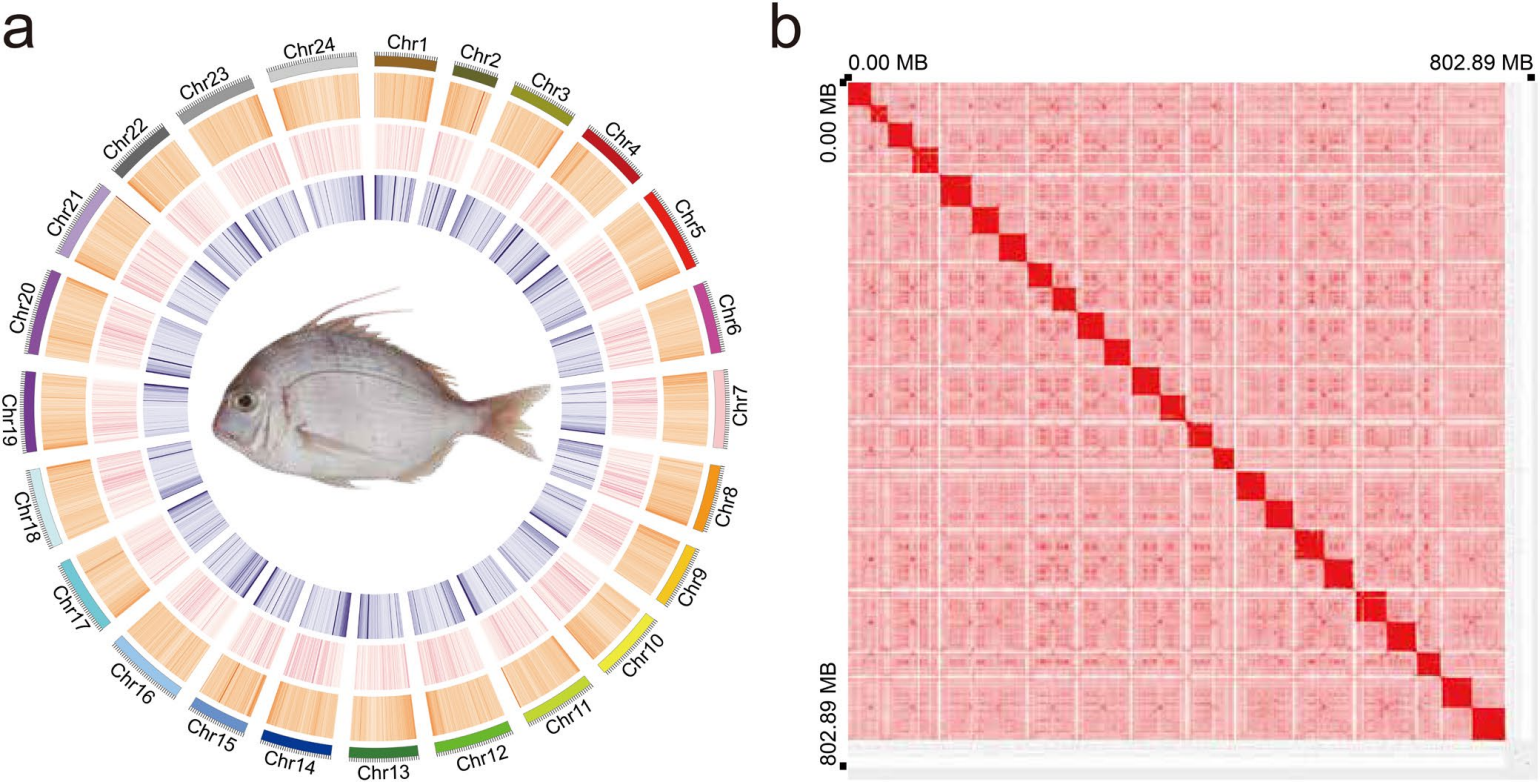
Genome assembly. (a) Circle plot of the genome features. From the inner to the outer layers: repeat element abundance (blue), gene abundance (pink), GC rate (yellow). (b) Heat map of interactive intensity between chromosome sequences anchored by Hi‐C.

In this study, we generated high‐throughput sequencing data for *E. cardinalis* , including Illumina (43.23 Gb, approximately 53.84 ×), PacBio HiFi (37.89 Gb, approximately 47.19 ×), Hi‐C (88.95 Gb, approximately 110.78 ×), and transcriptomic data (43.68 Gb). A genome survey (17‐kmer) estimated a size of 791.88 Mb, with 0.73% heterozygosity and 35% repeats (Table S1). HiFi reads were assembled and yield an 802.89 Mb genome (361 contigs, contig N50 = 11.18 Mb) (Table S1). Hi‐C reads were anchored into 24 chromosomes, producing an 802.94 Mb assembly (scaffold N50 = 32.29 Mb) (Table S1, Figure 2). And BUSCO evaluated genomic integrity at 98.9% (Figure S1). Repeat analysis identified 276.05 Mb (34.38% of genome) repetitive elements, including SINEs, LINEs, LTRs, and DNA transposons (Figure S2). Additionally, 6512 non‐coding RNAs (rRNAs, miRNAs, tRNAs, snRNAs) were annotated (Figure S2).

### 3.2 Gene Family Clustering and Phylogenetic Relationships

**FIGURE 3:**
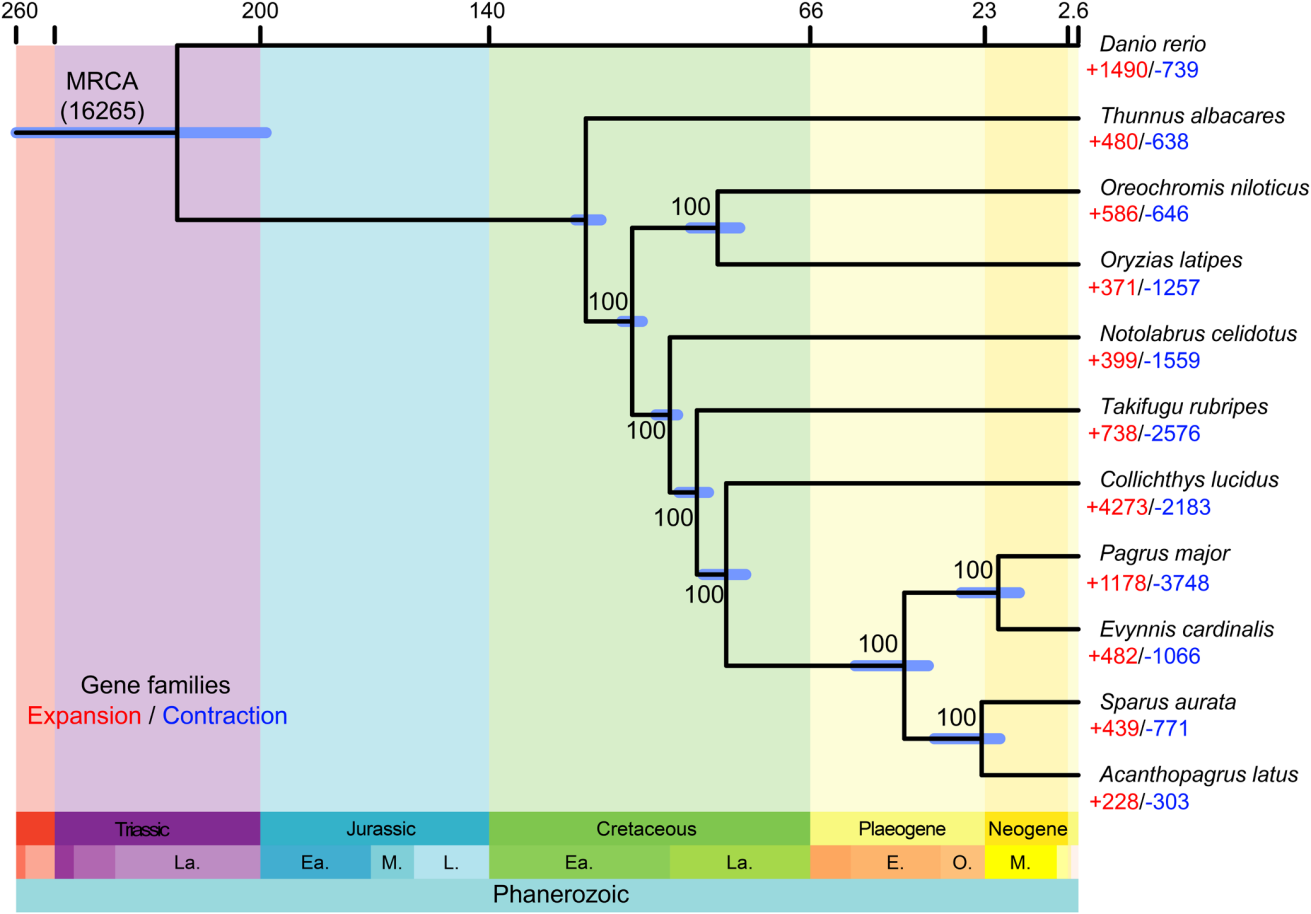
Phylogeny and gene family expansion and contraction analysis, gene family expansion (+) and contraction (−) are shown in red and blue, respectively.

We conducted gene family clustering of 273,036 protein‐coding genes from 11 fish species and yielded 22,324 gene families containing 260,118 genes, with 4056 identified as single‐copy orthologous families. Based on the 4056 single‐copy orthologous genes among the 11 bony species, we constructed a maximum likelihood (ML) phylogenetic tree. Divergence times between species were estimated based on TimeTree fossil calibration points. The phylogenetic tree revealed that all Sparidae species formed a well‐supported monophyletic clade, with *C. lucidus* identified as their closest relative (Figure 3). Within Sparidae, two major subclades were observed: one consisting of *S. aurata* and *A. latus* , and the other comprising *P. major* and *E. cardinalis* (Figure 3). Divergence time estimation suggested that Sparidae originated approximately 86.67 million years ago (Mya) (95% highest posterior density [HPD]: 81.85–91.40 Mya). The divergence between the two major Sparidae subclades occurred around 42.86 Mya (95% HPD: 36.94–54.95 Mya), while the split between *P. major* and *E. cardinalis* was estimated at approximately 19.71 Mya (95% HPD: 14.53–28.85 Mya) (Figure 3).

### 3.3 Gene Family Expansion and Contraction

Based on 16,265 gene families inferred from the most recent common ancestor (MRCA) of 11 fish species, we identified 482 expanded and 1066 contracted gene families in *E. cardinalis* , including 137 significantly expanded and 286 significantly contracted gene families.

**FIGURE 4:**
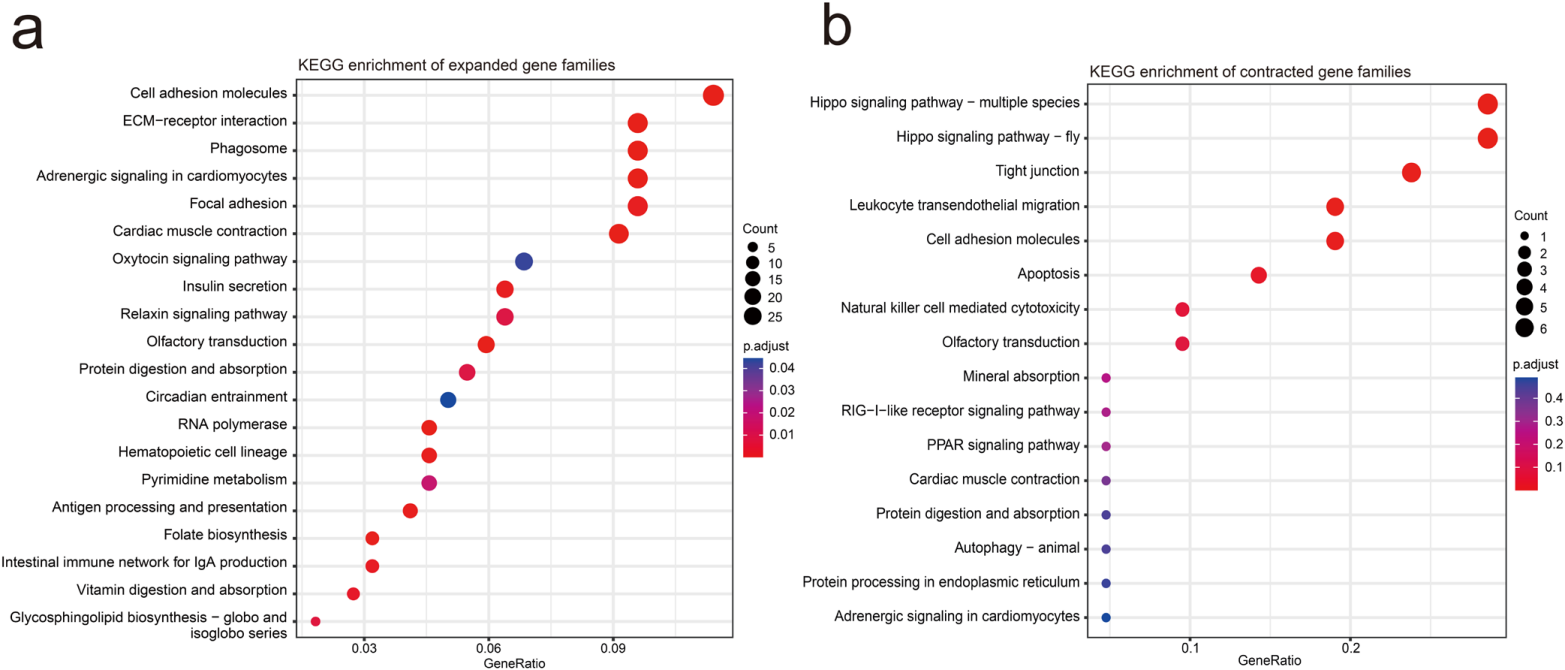
KEGG enrichment of the gene family. (a) The top 20 significantly enriched pathways in expanded gene families (*p* < 0.05). (b) The 16 enriched pathways in contracted gene families, and half of these had *p* < 0.05.

KEGG enrichment analysis revealed that the expanded gene families (Figure 4a, Table S2) were mainly concentrated in pathways related to cardiac function, including cardiac muscle contraction (k04260) and adrenergic signaling in cardiomyocytes (k04977), indicating a significant expansion trend of genes related to the circulatory system, which may help maintain continuous movement ability during the migration. The expanded gene families also enriched in olfactory transduction (k04740), suggesting that this species may have a stronger chemical perception ability to effectively find food, identify conspecifics, or avoid predators in benthic environment, thereby enhancing its ecological competitiveness. Furthermore, several immune‐related pathways were enriched, such as phagosome (k04145), antigen processing and presentation (k04612), and intestinal immune network for IgA production (k04672), indicating that their mucosal and innate immune systems may have been strengthened, thereby enhancing its survival capacity in nearshore environment with complex pathogenic pressures. In contrast, the contracted gene families (Figure 4b, Table S3) were mainly enriched in the Hippo signaling pathway (k04392), which is closely related to the regulation of organ size and the maintenance of cell homeostasis. These results suggest that, during its adaptation to benthic environments, *E. cardinalis* has prioritized the circulatory system, olfactory transduction, and immune functions rather than organ growth.

### 3.4 Positive Selection and Rapid Evolutionary Gene Identification

Comparative genomic analyses revealed that *E. cardinalis* and *P. major* exhibit faster evolutionary rates compared to other species (Figure S3). Positive selection, a key driver of adaptive evolution, facilitates the fix beneficial mutations during gene evolution. Using the PAML software, we identified a total of 608 PSGs and 399 REGs in *E. cardinalis* . Although significant pathway enrichment is largely absent due to weak genetic signals, KEGG pathway analysis still indicated that several PSGs and REGs are enriched in metabolic pathways related to energy production, including oxidative phosphorylation and the tricarboxylic acid (TCA) cycle (*DLD*, *SUCLG2*, *IDH3B*, and *ATP5F1*), which may enhance metabolic efficiency in *E. cardinalis* and is conducive to continuous movement during the migratory (Table S4). Additionally, several immune‐related genes, such as *SIKE1*, *TRAF4*, *PIN1*, *CHUK*, and *MAP3K7*, were under positive selection (Table S4), suggesting a potential role in dealing with the pressure of pathogenic microorganisms, which is essential for enhancing survival rates. Furthermore, sensory‐related genes such as *CPB1* and *P2RX3* were also identified among the PSGs and REGs (Table S4), implying that these genes may contribute to environmental sensing and behavioral adaptation in *E. cardinalis* , thereby supporting its survival in various ecological environments.

### 3.5 Population Structure Combined with Genomics and Machine Learning

**FIGURE 5:**
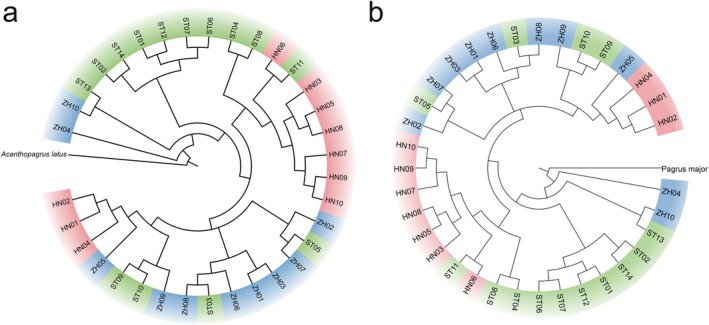
Population genetic structure based on (a) mitochondrial genome and (b) genome‐wide SNPs.

**FIGURE 6:**
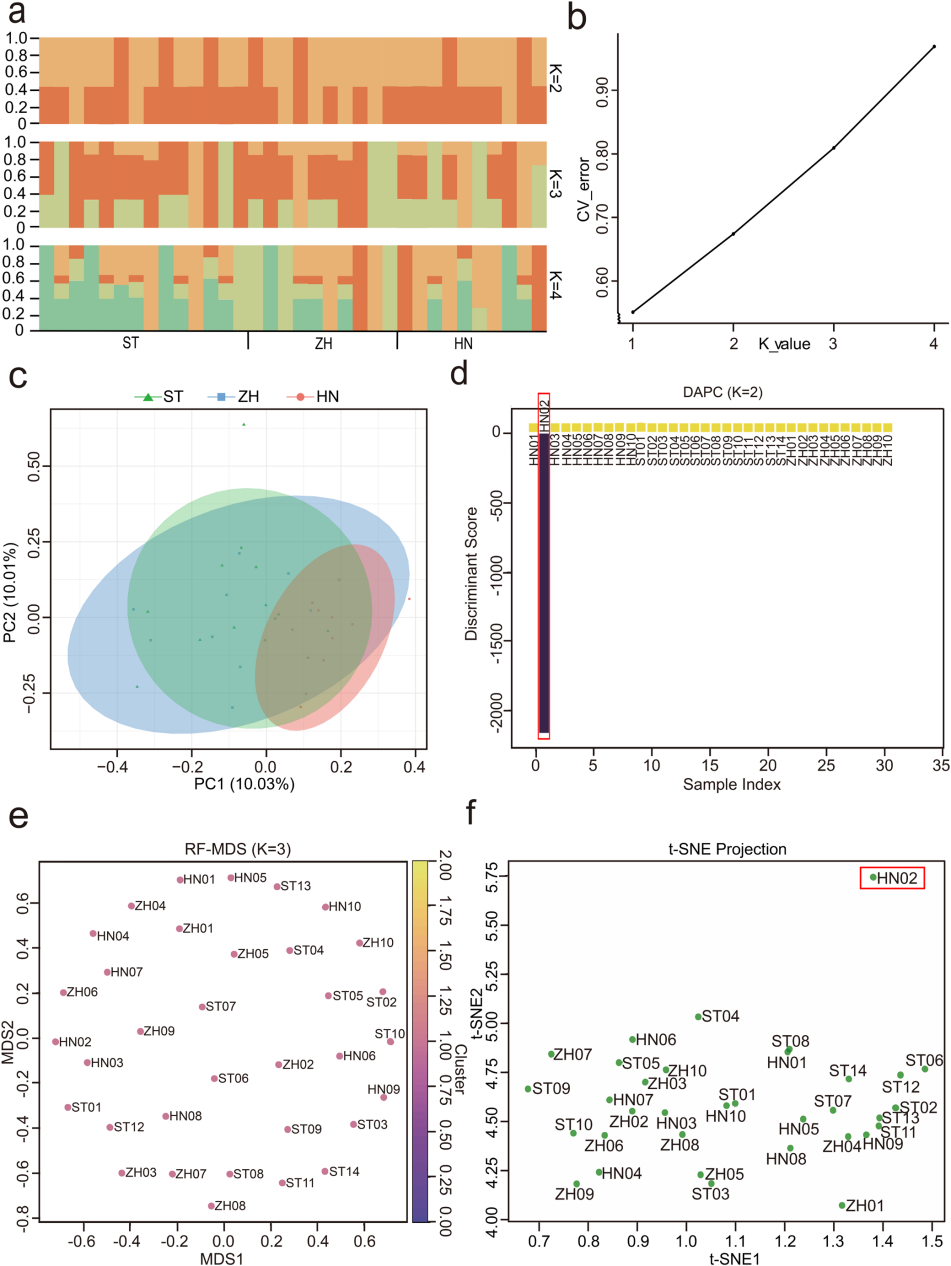
Population genetic structure analysis based on the genome‐wide SNPs. (a) Admixture analysis. (b) CV error values for different K values. (c) PCA analysis. (d) DAPC. (e) RF‐MDS. (f) t‐SNE analysis through machine learning methods.

Based on WGS data, a total of 52,699,042 SNPs were identified and 8,643,907 filtered SNPs were retained across 34 individuals of *E. cardinalis* . To investigate the population genetic structure, we first constructed a mitochondrial genomic phylogenetic tree (maximum likelihood, ML) using *A. latus* as an outgroup. The results revealed that individuals from the three geographical populations were intermixed without clear genetic differentiation (Figure 5a). Meanwhile, we conducted phylogenetic tree, PCA, and population structure analysis based on the high‐quality SNPs. Using *P. major* as an outgroup, a NJ tree was constructed based on genetic distance. The resulting phylogenetic tree showed that individuals from all three populations were mixed together, with no clear population clustering or separation (Figure 5b). Additionally, the Admixture analysis supported these findings, as the CV error increased with higher K values and reached its minimum at *K* = 1, suggesting the absence of detectable population structure (Figure 6a,b). Similarly, the PCA plot revealed no distinct clustering by population, indicating no apparent population structure (Figure 6c).

To further verify the accuracy of the above results, we adopted three complementary machine learning methods. The results obtained by the three methods are highly consistent in the overall trend. DAPC analysis identified that sample HN02 had a significantly deviated genetic background from its population, demonstrating potential genetic isolation characteristics (Figure 6d). The remaining 33 samples were highly clustered into a continuous and homogeneous group, and no obvious differentiation pattern based on geographical sources (ST, ZH, and HN) was observed (Figure 6d). RF‐MDS further supports this conclusion. Although this method divides into three clusters (*K* = 3), the samples of each geographical population show a highly mixed state in the clusters and do not show a spatial aggregation trend (Figure 6e). Combining the tolerance of random forests to variable noise, this result effectively rules out the possibility of pseudo‐clustering caused by geographical factors. The t‐SNE results provide supplementary evidence from the perspective of spatial structure. Except for HN02, the remaining samples show a continuous distribution in the two‐dimensional space, and no obvious population discontinuity was observed, demonstrating typical genetic homogeneity topological characteristics (Figure 6f). In conclusion, the three methods of DAPC, RF‐MDS, and t‐SNE complement each other and cross‐validate each other. None of them support the genetic subpopulation division based on geographical sampling points, indicating that there is no obvious genetic differentiation of this species among different geographic populations, presenting a highly homogeneous population structure.

### 3.6 Genetic Diversity and Adaptive Loci Identification

Based on the 8,643,907 filtered high‐quality SNPs, we also explored genetic diversity and population differentiation among three different geographical populations. The results showed that the average *Pi* value across the three populations was 3.06 × 10^−3^, with minimal differences among populations, indicating that nucleotide diversity levels were largely consistent across geographic groups. Pairwise *F*_*ST*_ values between populations were very low: 0.92 × 10^−3^ between the ST and ZH populations, 0.97 × 10^−3^ between the ZH and HN populations, and 1.06 × 10^−3^ between the ST and HN populations. All *F*_*ST*_ values were much lower than 0.05, suggesting low genetic differentiation among populations. Tajima's D values of the three populations were 0.49 (HN), 0.47 (ZH), and 0.67 (ST), respectively, indicating that there are some moderate‐frequency alleles, which may be due to the recent population bottleneck effect leading to the population contraction.

**FIGURE 7:**
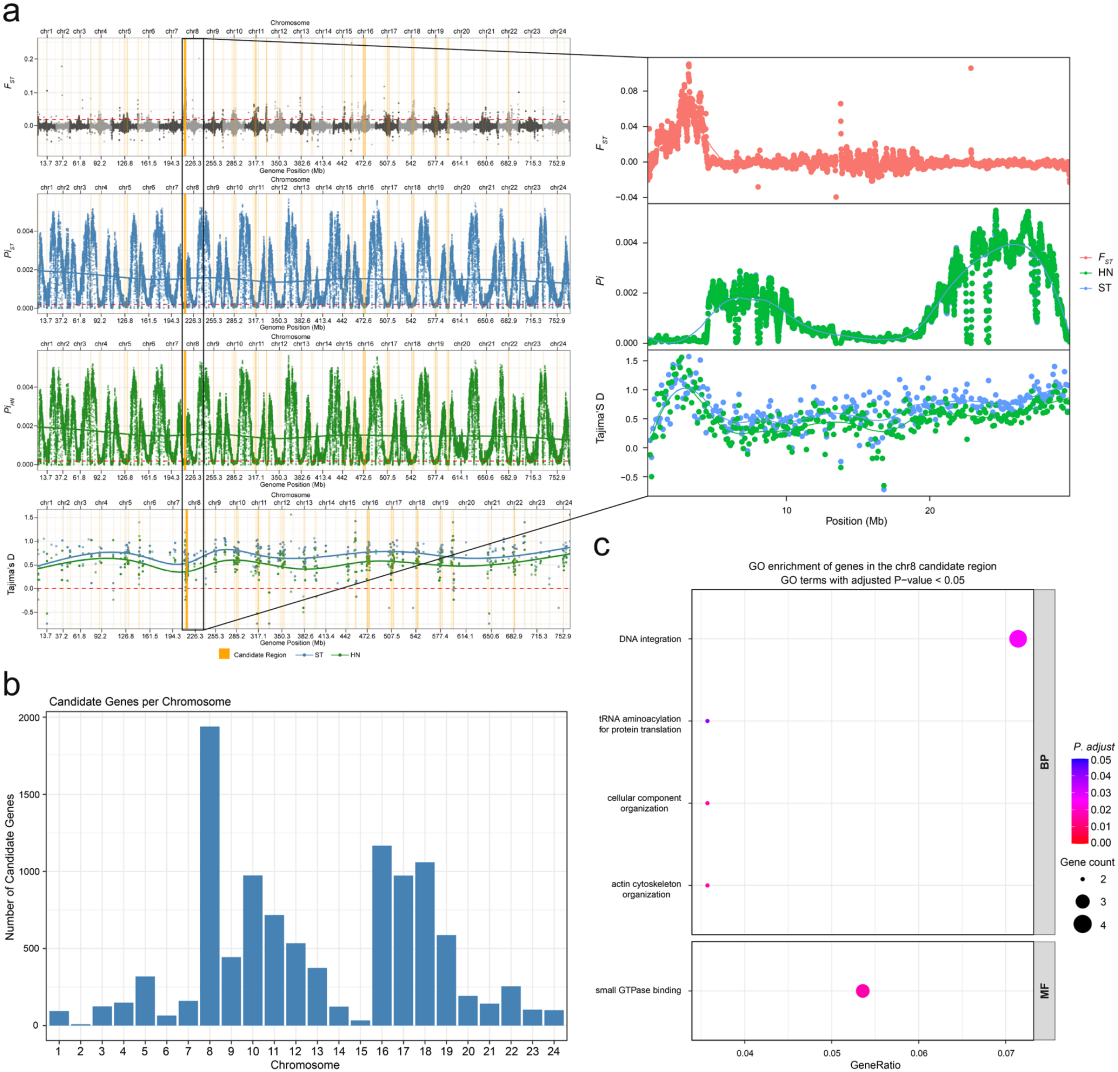
Potential differentiation‐associated region and GO enrichment on chromosome 8 between ST and HN populations. (a) Manhattan plots of *F*_*ST*_, Pi_ST, Pi_HN, and Tajima's *D* across all chromosomes, and the orange lines indicates candidate regions. The red box indicates the candidate region on chromosome 8, and the enlarged panel on the right shows the corresponding *F*_*ST*_, *Pi*, and Tajima’ *D* patterns across this region. (b) Distribution of candidate gene counts per chromosome. (c) GO enrichment of genes located within candidate regions on chromosome 8.

We further conducted a detailed analysis of genomic variations between ST and HN populations because they represent the greatest geographic differences and exhibit the highest pairwise *F*_*ST*_ values. Focusing on this contrast may increase sensitivity for detecting localized differentiation under heterogeneous nearshore conditions. We identified potential differentiation‐associated regions for adaptive evolution on chromosome 8, which also has the largest number of candidate genes (Figure 7a,b, Table S5). To further evaluate whether this region represented a robust differentiation signal rather than a relative outlier caused by stochastic fluctuations under the low genetic differentiation background, we compared *F*_*ST*_ values for the chromosome 8 candidate region with genome‐wide windows. The results showed that the *F*_*ST*_ for the chromosome 8 candidate region was significantly higher than that for the genome‐wide background. The average *F*_*ST*_ was 0.00942 for the candidate region, approximately 8.89‐fold higher than the genome‐wide average *F*_*ST*_ of 0.00106. The average *F*_*ST*_ of this region was higher than 92.38% of genome‐wide windows, and 15.24% and 10.43% of candidate region windows exceeded the genome‐wide 95th and 99th percentile thresholds, respectively. A Wilcoxon test further supported significantly higher *F*_*ST*_ in this region than in the genome‐wide background (*p* = 1.65 × 10^−20^). We also quantified nucleotide diversity differences between the ST and HN populations within the chromosome 8 candidate region. ST showed a statistically significant but very modest reduction in nucleotide diversity relative to HN, with a *Pi*_*HN*_/*Pi*_*ST*_ ratio of 1.004736 and an ROD value of 0.004714. Across sliding windows, 60.73% of windows showed lower *Pi* in ST than in HN, and the paired Wilcoxon test was significant (*p* = 2.30 × 10^−38^). These results indicate that nucleotide diversity in ST was slightly but consistently reduced across the candidate region, although the magnitude of reduction was small. Notably, Tajima's *D* values observed in this region were generally positive, which is not consistent with the classical hard selective sweep. Therefore, although the chromosome 8 candidate region showed elevated *F*_*ST*_ and a statistically detectable reduction in nucleotide diversity (*Pi*) in ST, we consider it a potential differentiation‐associated region rather than definitive evidence of directional selection.

GO enrichment analysis showed that genes in this chromosomal region were significantly enriched in a limited number of broad functional categories. We found terms are related to microtubule‐based movement (*KIF6*, *KIF7*), actin cytoskeletal organization (*DAAM2*, *DAAM1*), transmembrane transport (*KCNF1*, *KSNS3*, *SLC7A13*, *SLC2A1*), and actin binding (*DAAM2*, *DAAM1*), reflecting possible association with cytoskeletal organization and cellular structural processes (Figure 7c, Table S6). At the same time, multiple translation‐related functions were also identified, such as rRNA processing (*DIEXF*), tRNA aminoacylation (*IARS2*, *WARS*), ATP binding (*IARS2*, *KIF6*, *KIF7*, *ROCK2*, *MYO6*, *WARS*), indicating that this region may be associated with basic translation‐related processes and nucleotide‐binding functions (Figure 7c, Table S6). In addition, some terms related to DNA‐associated or nuclear cellular functions were observed, such as BRCA1‐A complex (BRE), nucleolus (*MYT1L*, *NOL10*, *OTX2*), and protein homodimerization activity (*KCNF1*, *KSNS3*), which may provide functional context for imply potential roles in cell cycle regulation and DNA repair (Figure 7c, Table S6). Overall, these findings indicate that ST and HN populations may exhibit divergence in cellular structure, energy metabolism, and DNA repair. It indicates that when adapt to different nearshore environments, they may develop distinctive adaptation strategies in certain regions of the genome and occur potential population differentiation.

## 4 Discussion

In this study, we generated the first chromosome‐level reference genome for *E. cardinalis* using PacBio HiFi and Hi‐C technologies, and reconstructed its evolutionary history. It is phylogenetically close to *P. major* , with divergence estimated at 19.71 Mya. Furthermore, by integrating WGS data from three geographically distinct populations in Chinese coastal waters, we found that there was no obvious genetic differentiation among the different geographic populations and that they were a single homogeneous population. However, we also found that there may be potential adaptive differentiation sites on chromosome 8.

Previous studies have indicated that the glacial–interglacial cycles have greatly influenced marine biodiversity (Lin, Wei, et al. 2023). Our divergence‐time estimates suggest that the separation of the two major Sparidae lineages occurred during a period of global warming and sea‐level rise (19.71 Mya, late Oligocene—early Miocene), which may have increased ecological opportunities and habitat availability, thus facilitating lineage expansion and diversification (Siqueira et al. 2023). Fish inhabiting these different environments develop unique adaptation abilities to respond to environmental stress; analyzing these adaptive evolutionary strategies can help us better understand their value in ecosystems and provide a theoretical foundation for biodiversity conservation and fishery resource management. Our analysis of the *E. cardinalis* genome revealed significant adaptive evolution in immune responses, energy metabolism, oxygen transport, and olfactory system.

Among the significantly expanded gene families, genes involved in cardiac muscle contraction and adrenergic signaling in cardiomyocytes were highly enriched. Previous studies have shown that adrenaline exposure can restore myocardial contraction rate under hypoxic conditions (Roberts and Syme 2018), suggesting that *E. cardinalis* may display enhanced cardiac function and hypoxia tolerance. And the improved efficiency of oxygen and nutrient transport may support greater locomotor activity and thus contribute to the species to migration in the ocean. In addition, we identified several key genes involved in oxidative phosphorylation and TCA cycle (*DLD*, *SUCLG2*, *IDH3B*, and *ATP5F1*) that experienced positive selection or rapid evolution. The TCA cycle is a central metabolic pathway for the oxidation of carbohydrates, lipids, and some amino acids, and it generates large amounts of ATP through oxidative phosphorylation (Bowtell et al. 2007). These evolutionary changes may enhance the energy metabolism of *E. cardinalis* , thereby improving its physical capability and endurance. This can also provide strong physiological support for the reproductive migration habits. Regarding immune function, several immune‐related signaling pathways were also identified in the significantly expanded gene families, and immune regulatory genes such as *PIN1*, *CHUK*, and *MAP3K7* exhibited signals of positive selection and rapid evolution. Similar variation in immune‐related gene families has also been observed in other species, such as the *Larimichthys crocea* (Wu et al. 2014) and the *Miichthys miiuy* (Xu et al. 2016). These demersal species primarily live in coastal benthic environments, which have greater microbial diversity, prokaryote and eukaryote organisms compared to pelagic zones (Ohore et al. 2022), and probably face more diverse pathogenic challenges. Thus, enhanced immune function is probably an adaptive response to the complex microenvironment in the benthos, providing competitive advantages for niche occupation and survival.

Genetic diversity and population structure of marine fish are critical for fisheries management and conservation. To clarify the population structure of *E. cardinalis* in Chinese coastal waters, we selected three populations (ST, ZH, and HN) inhabiting ecologically distinct environments. The Taiwan Strait, influenced by the Kuroshio Current, is characterized by higher water temperatures, salinity, and transparency (Song et al. 2016). The Pearl River is highly influenced by freshwater and rich in nutrients, with oversaturated levels of CH_4_, N_2_O, and CO_2_ (Chen‐Tung et al. 2008). In contrast, the Beibu Gulf is a semi‐closed bay recognized as a biodiversity hotspot in global marine ecosystems, with high water temperature and low Chl‐a concentration in summer due to southwest monsoon, and low water temperature and high Chl‐a concentration due to northeast monsoon in winter (Shen et al. 2018). In this study, analyses based on both mitochondrial genomes and genome‐wide SNPs failed to detect significant genetic differentiation among the three geographical populations of *E. cardinalis* , supporting it as a single genetic unit. This finding is consistent with multiple mitochondrial and nuclear gene‐based studies (Gong 2007; Bing Yang 2015; Xu et al. 2021).

Importantly, low levels of genetic differentiation do not imply a complete absence of local adaptation. Even under high gene flow, local environmental pressures (e.g., ocean currents, temperature gradients, or ecological barriers) may drive adaptive variation in species that is not reflected in the overall genome structure (Benestan et al. 2016; Andersson et al. 2023). Previous morphological evidence reported phenotypic differentiation between populations from Beibu Gulf and Taiwan Strait (Zhang and Cai 1983), seemingly inconsistent with the genome‐wide genetic homogeneity observed in our study. This inconsistency may be due to the fact that morphological characteristics are influenced by environmental conditions (e.g., temperature, salinity, food availability), which can induce phenotypic plasticity without corresponding genetic differentiation (Bertinetti and Torres‐Dowdall 2025).

In this study, although genome‐wide divergence was low in *E. cardinalis* , we detected several functional differentiations on chromosome 8 between ST and HN populations (e.g., microtubule‐based movement, ATP binding, protein synthesis, and DNA repair). The enrichment of genes related to microtubules (e.g., *KIF6*, *KIF7*) suggests that the organization and function of the cytoskeleton may play a key role in population adaptation. Microtubules are not only involved in intracellular transport but also influence cell shape and division, which could be crucial for the formation of morphological differences and adaptation (Dogterom and Koenderink 2019). Additionally, *FOSL2*, as a transcription factor, plays an essential role in the control of immune homeostasis and autoimmunity, while *OTX2* is primarily involved in regulating brain and eye development. These findings suggest that these genes may play a role in regulating the developmental differences between populations, particularly in shaping morphological traits (Renoux et al. 2020; Simeone 1998). Moreover, *SLC7A13*, which is involved in amino acid transport, significantly impacts cellular metabolism and growth and may be linked to nutritional requirements and metabolic adaptations between populations (Hushmandi et al. 2024). These findings in our study may serve as the molecular basis for the observed morphological differences between populations.

Similar patterns of “whole‐genome homogeneity with local differential” have also been observed in other marine fish species. Timm et al. (2024) found that despite the sablefish ( *Anoplopoma fimbria* ) exhibiting panmixia at the population level, significant signs of genetic differentiation were detected in two chromosomal inversion regions. This indicates that structural variations can maintain local genetic differences even in the presence of high gene flow. Similarly, Papa et al. (2022) noted that although the population genetic structure was nearly homogeneous in New Zealand terakihi ( *Nemadactylus macropterus* ), environmental association analysis revealed 55 adaptive loci associated with gradients in seawater temperature. These studies all suggest that, even in the context of genetic homogeneity, local genomic regions may still carry environmentally driven adaptive signals. Combining our molecular findings on chromosome 8 of *E. cardinalis* and existing research, it may reflect a similar process that, even under the influence of high gene flow, specific genomic regions are maintained in a state of functional differentiation due to selective pressures, phenotypic plasticity, or differences in chromosome structure.

Due to limitations in sample size and geographic coverage, coupled with the absence of direct genotype–phenotype associations, our study remains inadequate in detecting subtle population structure and adaptation traits. However, from a fisheries management perspective, the genome‐wide evidence in our study supports treating *E. cardinalis* along the Chinese coasts as a single management unit for stock assessment and conservation. And enhanced long‐term dynamic monitoring is still necessary to timely catch population differentiation signals driven by gene expression regulation. Previous studies have found substantial regional differences in resource abundance of this species, and its resources are highly vulnerable to overfishing (Xu et al. 2022). Furthermore, according to the research based on ecological models by Zhang et al. (2025), under current and future climate change conditions, the proportion and distribution pattern of suitable habitats in different sea areas of the northern South China Sea are significantly different, revealing the response differences of *E. cardinalis* to environmental factors (e.g., spatial distribution influenced by water depth and sea surface height). Thus, future studies should expand sampling across additional places and integrate ecological, environmental, and functional genomic approaches to monitor potential local adaptation and ensure the long‐term sustainability of management strategies.

Overall, we assembled the first chromosome‐level reference genome of *E. cardinalis* using HiFi and Hi‐C data. Through comparative genomic analysis, we identified a close phylogenetic relationship between *E. cardinalis* and *P. major* and revealed key signatures of adaptive evolution associated with its benthic coastal lifestyle. Furthermore, WGS of individuals from three geographic populations demonstrated a high level of genetic homogeneity among *E. cardinalis* populations along the Chinese coast. These findings provide valuable insights for the conservation and scientific management of this economically important species.

## Funding

This work was supported by the Key Program of the National Natural Science Foundation of China (42230413), the Guangdong Basic and Applied Basic Research Foundation (2024A1515011082), Guangdong Province Key Project (2023TX07A311), the Scientific Research Foundation for Talents, STU (NTF21026), the Science and Technology Plan Projects of Guangdong Province (2021B1212050025), and the Program for University Innovation Team of Guangdong Province (2022KCXTD008).

## Conflicts of Interest

The authors declare no conflicts of interest.

## Supporting information


**Figure S1:** BUSCO results of the assembled genome and proteins.
**Figure S2:** Repeat element types of the *E. cardinalis* genome.
**Figure S3:** Relative evolutionary rates of 11 fishes.

**Table S1:** The statistics of genome assembly of the *E. cardinalis* .
**Table S2:** KEGG enrichment of expanded gene families in *E. cardinalis* .
**Table S3:** KEGG enrichment of contracted gene families in *E. cardinalis* .
**Table S4:** KEGG enrichment of positively selected genes (PSGs) and rapidly evolving genes (REGs) in *E. cardinalis* .
**Table S5:** Distribution of candidate gene counts per chromosome.
**Table S6:** GO enrichment of genes located within candidate regions on chromosome 8.

## Data Availability

The data that support the findings of this study have been deposited into CNGB Sequence Archive (CNSA) of the China National GeneBank DataBase (CNGBdb) with accession number CNP0008071.
