# Supplementary material for: The Genetically Homogeneous Population Structure of Evynnis cardinalis Along the Chinese Coast Revealed by Whole Genome Sequencing
Source: Evol Appl. 2026 May 28;19(5):e70272. doi: 10.1111/eva.70272 (PMC13240142; doi:10.1111/eva.70272)
Supplement: Supplementary file 1 — Figure S1: BUSCO results of the assembled genome and proteins. Figure S2: Repeat element types of the E. cardinalis genome. Figure S3: Relative evolutionary rates of 11 fishes. [file EVA-19-e70272-s002.docx]

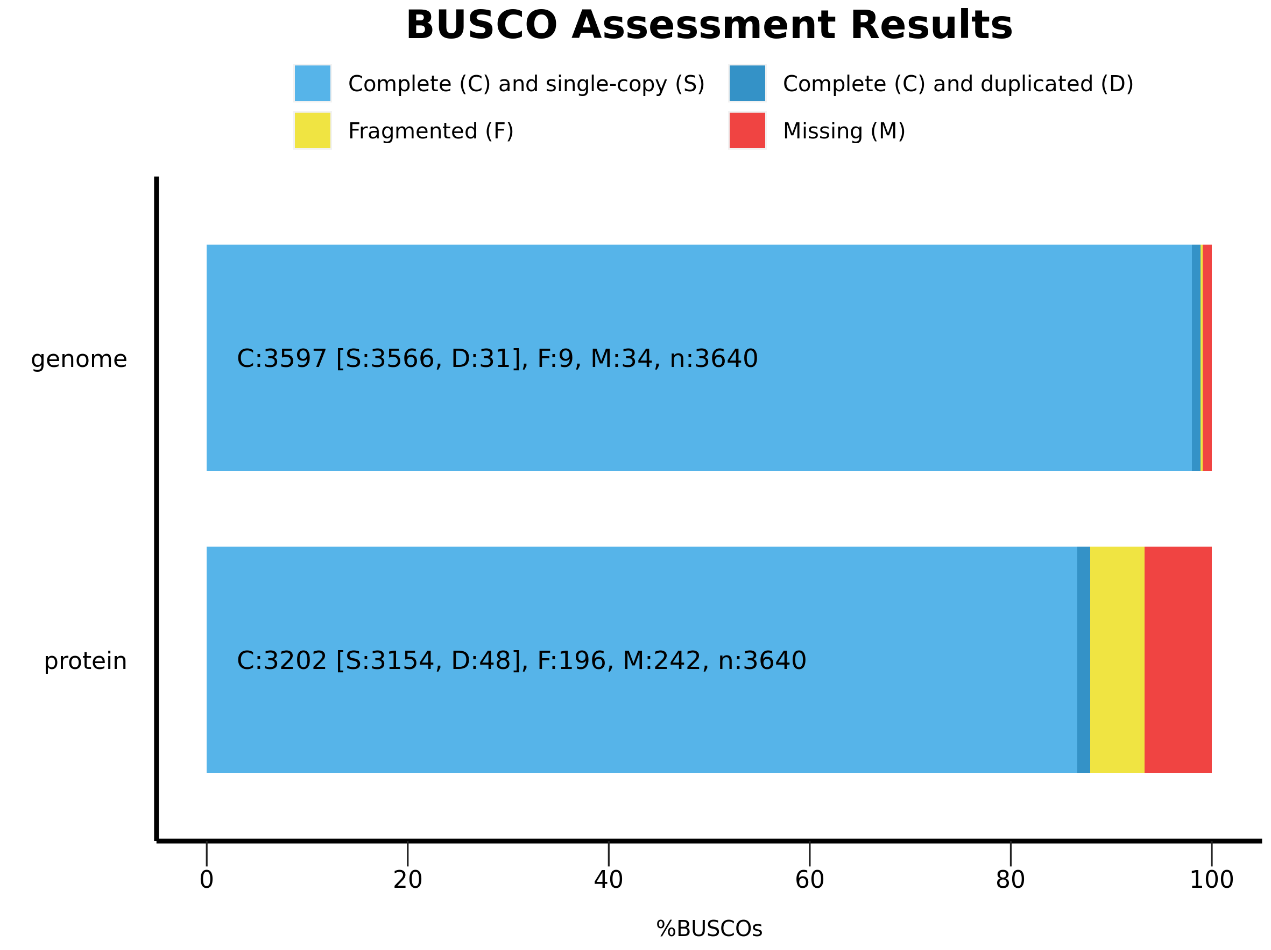
**Figure S1** BUSCO results of the assembled genome and proteins.

**
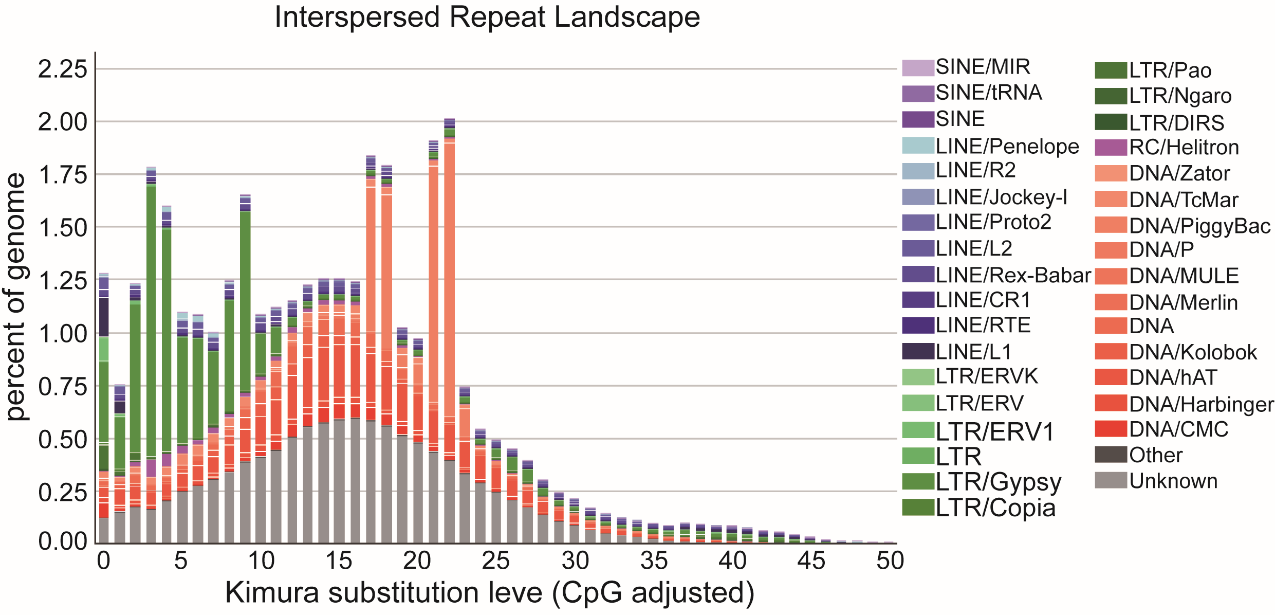
**

**Figure S2** Repeat element types of the *E. cardinalis* genome.


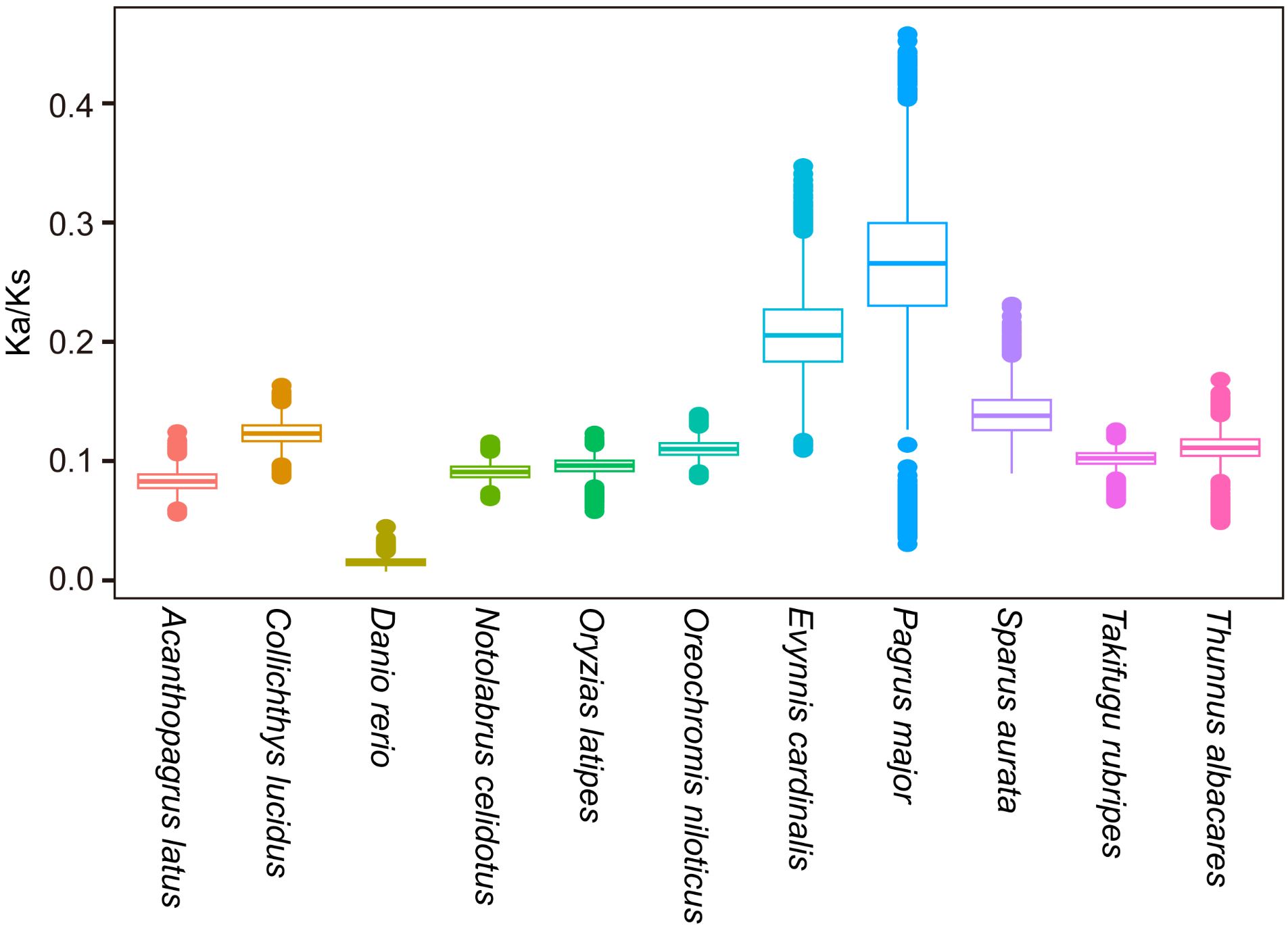


**Figure S3** Relative evolutionary rates of 11 fishes.
